# Correlation between mutans streptococci counts of parents and their children residing in Chandigarh, India

**DOI:** 10.4317/jced.51372

**Published:** 2014-07-01

**Authors:** Parampreet Pannu, H S. Chawla, Amrit Tewari, K. Gauba, Amrita Sujlana, Ramandeep S. Gambhir

**Affiliations:** 1 Professor and Head. Dept. of Pedodontics and Preventive Dentistry, Gian Sagar Dental, College and Hospital, Rajpura, Punjab; 2 Professor and Ex Head. Dept. of Pedodontics and Preventive Dentistry, OHSC, PGIMER, Chandigarh; 3 Professor and Director. Dept. of Pedodontics and Preventive Dentistry, OHSC, PGIMER, Chandigarh; 4 Dental Consultant. Dept. of Pedodontics and Preventive Dentistry. Sukh-Iqbal Dental Clinic, Chandigarh; 5 Sr. Lecturer. Dept. of Public Health Dentistry, Gian Sagar Dental College and Hospital, Rajpura, Punjab

## Abstract

Objectives: To determine the prevalence of Mutans Streptococci (MS) in children and to evaluate the relationship between the salivary levels of MS in children and their parents. Material and Methods: 100 children aged 3-6 years along with their parents (100 mothers and fathers each) were included in the study. The children were segregated depending upon their age and level of caries experience. The children were examined clinically on an ordinary chair in natural daylight using the Møller’s index criteria. Assays for Mutans Streptococci (MS) were done for both the children and their parents using the Dentocult SM strip mutans test. Collected data was assessed using SPSS software. Results: The overall prevalence of MS in the children was 83%. A statistically significant correlation (p<0.05) was observed between salivary MS counts of children and their mothers. No significant correlation was seen with the fathers. Conclusions: The findings conclude that if primary caregivers harbour high levels of MS in their saliva, it is likely that their children will have the same.

** Key words:**Mutans streptococci, parents, children, dentocult SM kits.

## Introduction

The World Health Organization [WHO] fact sheet on oral health abundantly authenticates the worldwide prevalence of dental caries (1). The ubiquity of this disease is an outcome of its highly infectious nature. Even though dental caries has a multifactorial etiology, the specific factor which results in its high rate of transmissibility is the microbial component i.e. Mutans Streptococci [MS]. Mutans Streptococci, specifically Streptococcus mutans and Streptococcus sobrinus, have been identified as the primary microorganisms implicated in the initiation and development of dental caries. The outmoded concept of MS acquisition by children from earlier studies stated that these microorganism are acquired from mothers only after primary tooth eruption (2). Today it is a known fact that MS can colonize the mouths of pre dentate children through both vertical as well as horizontal transmission (3,4). Early acquisition of MS is considered to be a major risk factor in the development of Early Childhood Caries [ECC] (5) as well as future caries experience (6,7).

The early years of a child’s life are primarily oriented around parents. Mothers, being the primary caregivers, have a close association with their children, and in doing so, are the main source of infectious micro flora to them (8). The nascent stages of childhood are largely spent ensconced in the mothers shelter, but with nuclear families fast emerging, fathers today share equal responsibilities. Relationship between the strains of MS in fathers and infants has been studied (9,10) but only one study found a similarity of MS strains in 31.4% of children with that of their fathers (11).

The present study was carried out involving parents and their children of Chandigarh, India [i] to determine the prevalence of MS in children and [ii] to evaluate the relationship between the salivary levels of MS of children and that of their parents.

## Material and Methods

The present study was conducted in the Department of Pedodontics and Preventive Dentistry at Postgraduate Institute of Medical Education and Research [PGIMER], Chandigarh. The study protocol was approved by the ethics committee of PGIMER, Chandigarh. A written informed consent was obtained from the selected participants.

- Study Subjects

One hundred children, aged between 3-6 years, were selected from various schools of Chandigarh using a systematic random sampling technique. After obtaining contact information from the school records, parents were contacted by telephone to explain the purpose of the study and seek permission to visit them. They were also instructed not to eat/drink, brush their teeth, use a mouth wash or smoke one hour prior to their scheduled appointment. The selected households were visited by one of the authors [PP] and written consent was obtained from the parents prior to participation in the study. Care was taken to include healthy children free of any systemic disease.

The study cohort [n = 100] was divided into two groups depending upon their age; Group 1 aged < 4 ½ years [n = 51] and Group 2 aged > 4 ½ years [n = 49]. This was done to assess if a change from kindergarten to primary school affected the MS acquisition. Additionally the children were also stratified on the basis of their caries status into a low dental caries group with [dmfs score 0-3; n=51] and a high dental caries group [dmfs score > 3; n = 49].

- Clinical examination

Assessment of dental caries was done performed during the home visit with the participants seated on an ordinary chair in natural daylight, facing away from direct sunlight, using a mouth mirror and a blunt probe. Møller’s index (12) for dental caries recordings was used to record dmft and dmfs as it records the initial carious lesions and initiation of dental caries is associated with the presence of S. mutans. The recording procedure was standardized by repeated sessions of calibrations between the examiner and one of supervisors. Taking into consideration all the differences and agreements, weighted kappa score was calculated to be between 0.8 and 0.95 which shows good intra-examiner reliability.

- SM assays

The Dentocult SM strip mutans test was used for estimation of salivary levels of MS. Each participant was given a paraffin pellet [provided in the kit], to be chewed upon for one minute and was instructed to swallow the stimulated saliva after removal of the pellet. A Dentocult SM Strip was turned around ten times in the participant’s mouth to contaminate it with saliva. Excess saliva was then removed by withdrawing the strip through closed lips. The contaminated strip was immediately transferred into a glass vial containing 6 ml of liquid MSB agar medium which is a selective culture broth containing mitis salivarius agar with sucrose, bacitracin and potassium tellurite. The vials containing Dentocult SM strips were coded and transported within an hour of collection to the laboratory in an upright position. In the laboratory, the vials were given a secondary code by one of the authors in order to make it a blind study. The screw cap of the vials was opened by quarter of a turn so as to release any gas which could form during incubation. The vials were incubated in 95% of N2 , 5% CO2 at 37ºC for 48 hours to allow growth of visible colonies on the strips. After the incubation period the strips were carefully removed from the vials with the help of tweezers and air dried. The colony density on the strips was compared with the standard interpretation chart [provided by the manufacturer in the kit] and the grading of the strips was done as score 0, 1, 2 or 3 by observing them with the naked eye. After the recording, the strips were packed in plastic bags and sent for incineration.

- Statistical methods

The recorded data was transferred to an excel sheet and statistical analysis was carried out using Statistical Package for Social Sciences [SPSS Inc., Chicago, IL, version 13.0 for Windows]. Descriptive statistics were calculated and Chi-square test was used to find association between variable. Pearson’s Correlation Coefficient was also used to correlate MS count of children with their parents [r-value]. The difference in percentage prevalence of MS between the two groups was analyzed using Z-test [r value]. p value < 0.05 was considered statistically significant.

## Results

A total of 100 children aged between 3-6 years were examined and the overall prevalence of MS was observed to be 83% in them ( Table 1). The prevalence of MS was 80.36% in Group 1 as compared to 85.95% in Group 2, thereby showing an increase in the MS count with increasing age but this increase was not found to be statistically significant. Another result to be acknowledged is that the number of children having MS count of >106 CFU/ml of saliva [Score 3] increased from 13.70% to 20.41% with increasing age years ( Table 1). When analyzing the distribution of children according to their MS scores, majority of the children showed a score of 2.

**Table 1:**
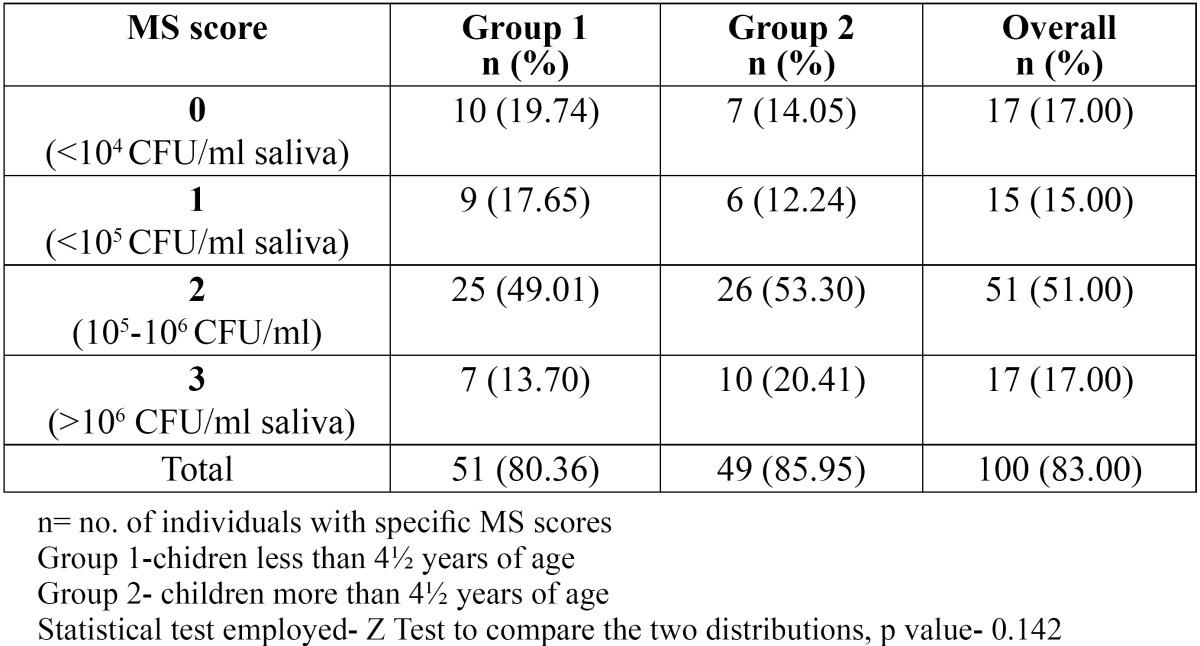
Frequency distribution of children as per their age and salivary MS scores.

 Table 2 correlates the salivary MS counts of children with their mean dmft/dmfs scores using Pearson’s Correlation Coefficient. It was observed that there was a simultaneous increase in the MS score and the mean dmft/dmfs score, thereby showing an increase in caries severity with an increase in MS counts [r value-0.326]. The overall percentage of children with detectable levels of MS in saliva was 68.70 % and 97.95 % in the low caries and high caries groups, respectively.

**Table 2:**
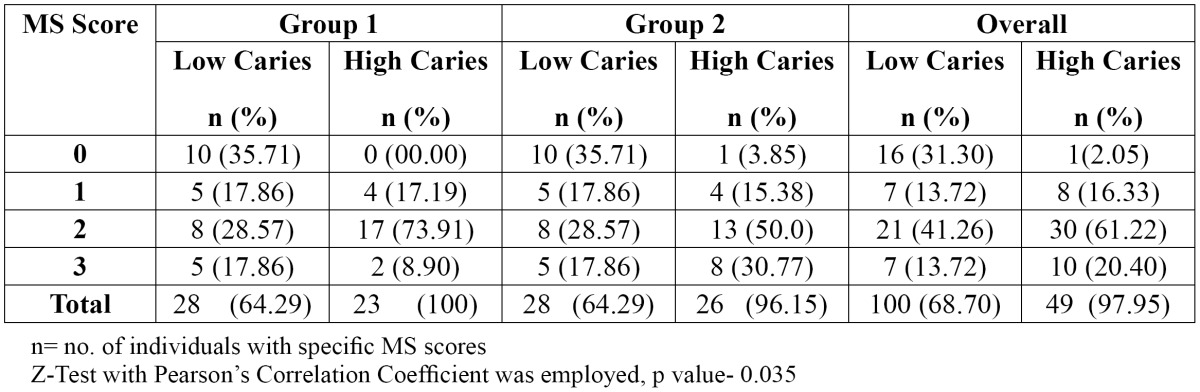
Association between dental caries of children and their salivary MS counts.

On comparing salivary MS counts of parents and their children belonging to the two groups based on their caries status, no statistically significant relationship was observed. But when the salivary MS counts of children in Group 2 were compared with that of their mothers, using Pearson’s correlation coefficient, chi-square test and weighted kappa, ( Table 3) a statistically significant relationship [p< 0.05] was noticed [Pearson’s Correlation Coefficient-0.4229] i.e. 37 mothers of 49 children aged 5-6 years had MS score of more than 105 CFU / ml of saliva and 30 children of theirs had similar high scores, whereas no such pattern was observed in 3-4 years age group. Chi-square test and Pearson’s Correlation Coefficient [r value-0.3241] revealed no significant correlation between the salivary MS count of fathers and their children [p>0.05] ( Table 4).

**Table 3:**
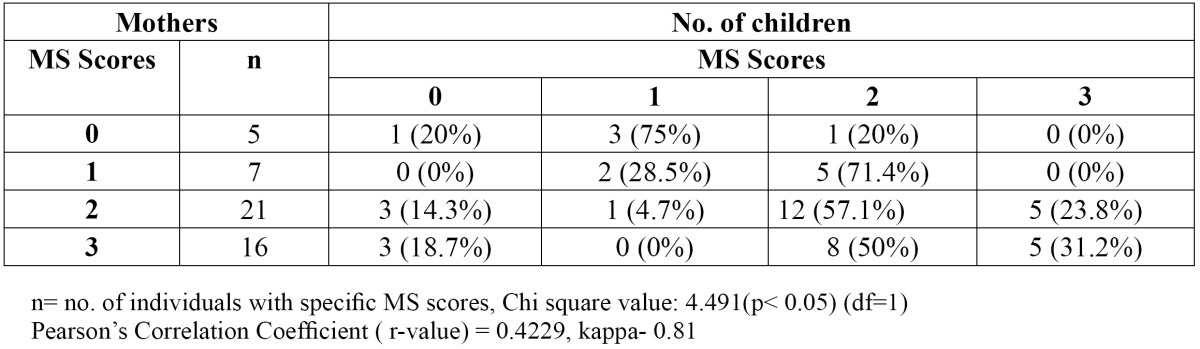
Association between salivary MS counts of children aged 5-6 years and their mothers.

**Table 4:**
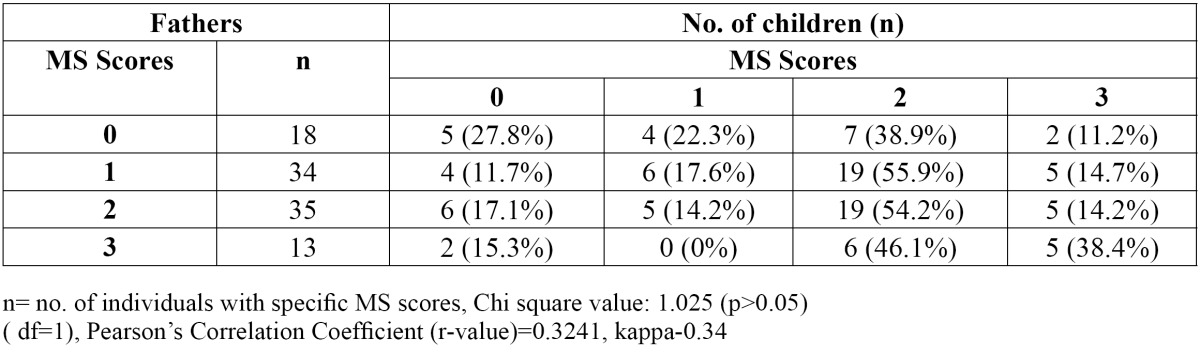
Association between salivary MS counts of children aged 5-6 years and their fathers.

## Discussion

Dental caries is a transmissible, pathological infectious disease that decalcifies the hard tissues of the teeth. Various studies have reported a strong positive association between MS and dental caries in humans (4,5). The present study was carried out on a total of 100 children aged between 3 to 6 years. This age group was chosen because the eruption of the deciduous teeth is usually completed by 3 years of age and the frequency of detection of MS increases with eruption of teeth.

According to some study reports, prevalence of MS was 46.3% in 4 to 5 year old children (7). In our study the overall prevalence of MS was found to be 83% in 3 to 6 year old children, being 80.39 % in 3 to 4 year old and 85.95 % in 5 to 6 year old children. According to reports of another study carried out in preschool children, the prevalence of MS was found to be 61.7% (13). The isolation frequency of MS in the present study is higher as compared to other studies (7,14). The possible reason for this finding may be that in the present study the decayed [d] component comprised 99.04% of the dmft, whereas in other study it was 32% in 5 year old children (7).

In our study a much higher prevalence of MS was observed i.e. 97.95%, in high dental caries group, as compared to 68.70% in the low dental caries group. This finding again establishes a known fact that MS tend to colonize carious or restored tooth surfaces more than sound tooth surfaces. Results of a study conducted in Sweden showed that Early-colonised children, had higher salivary MS levels and DFS than later MS-colonised children (15). The results indicate that it is possible to remain negative for MS into adulthood if non-colonised at an early age and this affects the development of carious lesions. According to some other study reports, MS was detected in 94% of children having ECC (16). Restored surfaces also tend to be more colonized by MS than sound surfaces, except for occlusal surfaces.

A significantly positive association was observed between the mothers having high salivary MS counts and that of their children aged 5-6 [p<0.05]. This may be explained by the fact that children are less heterogeneous than mothers when taking into consideration the multiple strains of MS in their oral cavity. As stated by Caufield *et al.*, children may continue to acquire the entire repertoire of oral microbiota as they grow, which may explain the increasing correlation seen in our study (17). On the other hand, the significant association seen in Group 2 reinforced the fact that the transmissibility of micro organisms is dependent upon the concentration of micro-organisms in the inoculums. Therefore, a high salivary concentration of MS of the mother will increase the chances of her transmitting the bacteria to her children. Several cross-sectional studies have also reported a positive correlation between salivary MS counts of children and their mothers (18-23).

We did not observe any correlation between the MS counts of the father and their children. A study carried out in Swedish families using chromosomal DNA fingerprinting has also shown similar genotypes between mot-her-child pairs whereas, but such an association was not seen in the father-child pairs (24). Unlike this study, a study carried out in a Turkish population analyzing the genotypic strains of MS in parent-child pairs have shown a significant correlation in both mother-child as well as father-child pairs (22). This variation in the results from different communities may have resulted from the difference in their child rearing practices.

A major disadvantage of our study is that we have not use intraoral lighting and additional diagnostic aids like bitewing radiographs to assess interproximal lesions which were hidden from direct vision. This might have significantly affected the caries assessment. Another limitation of our study is that we have not carried out genotype mapping of the MS strains, which may have further shown the intra-familial transfer of MS. We plan to further work on these lines and also research child rearing practices which may increase chances of MS transmission.

## Conclusions

Our study highlights the fact that if primary caregivers harbor high levels of MS in their saliva, it is likely that their children would also have high levels of MS colonization. Hence there remains a need for parent counseling and motivation, so that they not only maintain their own oral hygiene but also make a conscious effort to avoid transmission and colonization of these cariogenic bacteria to their children.
